# An improved synthesis of 1,3,5-triaryl-2-pyrazolines in acetic acid aqueous solution under ultrasound irradiation

**DOI:** 10.1186/1860-5397-3-13

**Published:** 2007-03-21

**Authors:** Ji-Tai Li, Xiao-Hui Zhang, Zhi-Ping Lin

**Affiliations:** 1 College of Chemistry and Environmental Science, Hebei University; Key Laboratory of Analytical Science and Technology of Hebei Province, Baoding 071002, P. R. China

## Abstract

**Background:**

Pyrazoline derivatives have been found to possess a broad spectrum of biological activities. Among various pyrazoline derivatives, 2-pyrazolines seem to be the most frequently studied. A variety of methods have been reported for the preparation of this class of compound. However, in spite of their potential utility, some of the reported methods suffer from drawbacks such as long reaction times, cumbersome product isolation procedures and environmental concerns. Organic reactions in aqueous media have attracted increasing interest recently because of environmental issues and the understanding of biochemical processes. Ultrasound has increasingly been used in organic synthesis in the last three decades. A large number of organic reactions can be carried out in higher yields, shorter reaction time or milder conditions under ultrasound irradiation.

**Results:**

Preparation of a series of 1,3,5-triaryl-2-pyrazolines through the reaction of chalcones and phenylhydrazine hydrochloride was carried out in 83–96% yield within 1.5–2 h in sodium acetate-acetic acid aqueous solution under ultrasound irradiation.

**Conclusion:**

We have described a practical and convenient procedure for the synthesis of 1,3,5-triaryl-2-pyrazolines in sodium acetate-acetic acid aqueous solution at room temperature under ultrasound irradiation.

## Background

Pyrazoline derivatives have been found to possess a broad spectrum of biological activities such as tranquillizing, muscle relaxant, psychoanaleptic, anticonvulsant, antihypertensive, and antidepressant activities. [1–6] Among various pyrazoline derivatives, 2-pyrazolines seem to be the most frequently studied pyrazoline type compounds. A variety of methods have been reported for the preparation of this class of compounds. After the pioneering work of Fischer and Knoevenagel in the 19th century, the reaction of α,β-unsaturated aldehydes and ketones with phenylhydrazine in acetic acid by refluxing became one of the most popular methods for the preparation of 2-pyrazolines. [7] In 1998, Powers *et al*. [8] reported that the reaction of chalcones and phenylhydrazine hydrochloride in the presence of sodium hydroxide was carried out in the absolute ethanol at 70°C, but there is a disadvantage due to longer the reaction time (8 h). In 2005, the synthesis of 3,5-diaryl-2-pyrazolines by the reaction of chlorochalcones with phenylhydrazine in acetic acid by refluxing for 3 h was reported by Levai, [7] yet the ratio of chlorochalcones and phenylhydrazine was 1:5. These reaction conditions suffer from economic and environmental concerns. Recently, K_2_CO_3_-mediated microwave irradiation has been shown to be an efficient method for the synthesis of pyrazolines. [9]

The recent interest in green chemistry has posed a new challenge for organic synthesis in that new reaction conditions need to be found which reduce the emission of volatile organic solvents and the use of hazardous toxic chemicals. Organic reactions in aqueous media have attracted increasing interest currently because of environmental issues and the understanding of biochemical processes. As a reaction solvent, water offers many practical and economic advantages including low cost, safe handling and environmental compatibility. Recently, many organic reactions in aqueous media have been described in the literature. [10]

**Scheme 1:**
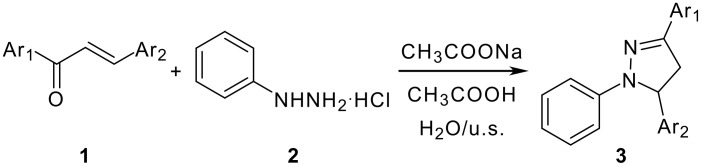
Synthesis of 1,3,5-triaryl-2-pyrazolines.

Ultrasound has increasingly been used in organic synthesis in the last three decades. Compared with traditional methods, the procedure is more convenient and can be carried out in higher yields, shorter reaction time or milder conditions under ultrasound irradiation. [11–13] Continuing our investigations on the application of ultrasound in organic synthesis, we wish to report an efficient and practical procedure for the synthesis of 1,3,5-triaryl-2-pyrazolines with chalcones and phenylhydrazine hydrochloride in sodium acetate-acetic acid aqueous solution under ultrasound irradiation (Scheme 1).

## Results and discussion

The effect of the reaction conditions on the reaction of chalcones and phenylhydrazine hydrochloride under ultrasound irradiation was summarized in Table 1. When the molar ratio of chalcones(**1**):phenylhydrazine hydrochloride(**2**) was 1:1, the yield of 1,3,5-triphenylpyrazoline obtained was 76% (Table 1, Entry **a**). By increasing the molar ratio to 1:2, and 1:3 the yields of **3a** increased to 82% and 89% respectively (Table 1, Entry **b**, **c**). The results showed that changing the molar ratio of **1:2** had a significant effect on the yield, and the optimum molar ratio of chalcone: phenylhydrazine was 1:3. The important discovery was that in the present of sodium acetate in acetate acid aqueous the yield of pyrazolines can be increased, it may be that sodium acetate is in favor of release of phenylhydrazine from phenylhydrazine hydrochloride. When the molar amount of sodium acetate increased from 0.15 to 0.2 and 0.3, the yield of pyrazoline decreased from 96% to 95% and 92% respectively (Table 1, Entry **d**, **e**, **f**). So the reaction conditions we chose were: the molar ratio of chalcone: phenylhydrazine: sodium acetate was 1:3:0.15.

**Table 1:** Effect of reaction condition on synthesis of 1,3,5-triphenyl-2-pyrazoline ^a^

**Entry**	**Molar ratio of 1/2/NaAc**	**Frequency (kHz)**	**Time (h)**	**Yield (%)**

**a**	1:1:0	25	2	76
**b**	1:2:0	25	2	82
**c**	1:3:0	25	2	89
**d**	1:3:0.15	25	2	96
**e**	1:3:0.2	25	2	95
**f**	1:3:0.3	25	2	92
**g**	1:3:0.15.	40	2	90
**h**	1:3:0.15	59	2	85
**i**	1:3:0.15	Stir^b^	4	76

^a^ Reaction temperature: 28–32°C, substrate: PhCOCH = CHPh, CH_3_COOH/H_2_O = 2/1(V/V). ^b^ Stirred without ultrasound irradiation.

In order to verify the effect of ultrasound irradiation, in the absence of ultrasound, we have performed the reaction of chalcone with phenylhydrazine hydrochloride by refluxing at 108°C for 4 h. The yield of pyrazoline was 76% (Table 1, Entry **i**). While under ultrasound irradiation, the reaction can be completed within 2 h in 96% yield at room temperature (Table 1, Entry **d**). It was clear that the ultrasound could accelerate the reaction of chalcone and phenylhydrazine hydrochloride.

We also monitored the effect of different irradiation frequencies on the reaction. When the frequency was 25 kHz, the yield of pyrazoline was 96% (Table 1, Entry **d**) within 2 h. Under 40 kHz and 59 kHz irradiation, the yield of pyrazoline was 90% and 85% respectively (Table 1, Entry **g, h**). It seems that the lower frequency of ultrasound irradiation can improve the yield of pyrazoline. It is possible that as the ultrasonic frequency is increased, the production of cavitation in liquids decreases. [11]

From the results above, the optimum reaction conditions was chosen: chalcone (**1**, 2 mmol), phenylhydrazine hydrochloride (**2**, 6 mmol), sodium acetate (0.3 mmol). Under this reaction system, a series of experiments for synthesis of 1,3,5-triphenyl-2-pyrazolines under 25 kHz ultrasound irradiation were performed. The results are summarized in Table 2.

**Table 2:** Synthesis of 1,3,5-triaryl-2-pyrazolines in the NaAc-HAc aqueous under ultrasound irradiation*

**Entry**	**Ar**_1_	**Ar**_2_	**T (°C)**	**Time (h)**	**Yield (%)**	**M.P. (°C) [lit.]**

**a**	C_6_H_5_	4-CH_3_OC_6_H_4_	28–33	1.5	96	110–112
**b**	C_6_H_5_	4-CH_3_C_6_H_4_	29–33	2	88	128–130
**c**	C_6_H_5_	C_6_H_5_	32–36	2	96	134–135(134–135) [9]
**d**	C_6_H_5_	4-ClC_6_H_4_	29–33	2	86	135–136(133–134) [7]
**e**	C_6_H_5_	3-ClC_6_H_4_	29–33	2	83	134–136
**f**	C_6_H_5_	2-ClC_6_H_4_	28–33	2	85	134–135(135–136) [7]
**g**	C_6_H_5_	3-BrC_6_H_4_	29–33	2	83	141–143
**h**	C_6_H_5_	4-O_2_NC_6_H_4_	28–34	3	trace	
**i**	4-ClC_6_H_4_	C_6_H_5_	28–33	2	85	143–145
**j**	3-O_2_NC_6_H_4_	C_6_H_5_	28–33	3	trace	

* The preparation of chalcones was referred to [14]

**Scheme 2:**
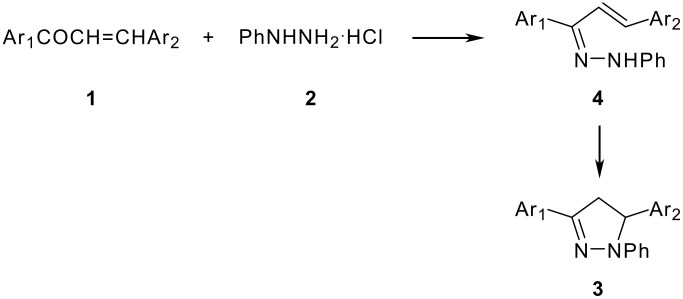
The mechanism of 1,3,5-triarylpyrazoline formation.

The following sequence of reaction appears to afford a satisfactory explanation of the mode of formation of the products (Scheme 2). This reaction involves the initial formation of an arylhydrazone with subsequent attack of nitrogen upon the carbon-carbon double bond. Condensations involving similar systems have been run in alcoholic hydrochloric acid. [15] However, it should point that the formation of intermediate (Scheme 2, **4**) through cyclization of the arylhydrazone has not been observed in the reaction.

## Conclusion

In summary, we have described a practical and convenient procedure for the synthesis of 1,3,5-triaryl-2-pyrazolines in sodium acetate-acetic acid aqueous solution at room temperature under ultrasound irradiation.

## Experimental section

[See Supporting Information File 1]

## Supporting Information

File 1 Experimental Section. Experimental detail data which includes experimental detail of the spectral instruments, synthesis of 1,3,5-triaryl-2-pyrazolines,^1^H NMR, ^13^C, IR and elemental analysis data along with ultrasonic instrument.
