# Supplementary material for: An improved synthesis of 1,3,5-triaryl-2-pyrazolines in acetic acid aqueous solution under ultrasound irradiation
Source: Beilstein J Org Chem. 2007 Mar 21;3:13. doi: 10.1186/1860-5397-3-13 (PMC1847524; doi:10.1186/1860-5397-3-13)
Supplement: File 1 — Experimental Section. Experimental detail data which includes experimental detail of the spectral instruments, synthesis of 1,3,5-triaryl-2-pyrazolines,1H NMR, 13C, IR and elemental analysis data along with ultrasonic instrument. [file Beilstein_J_Org_Chem-03-13-s001.doc]

Experimental Section

General

Liquid substrates were distilled prior to use. Melting points were uncorrected. ^1^H NMR and ^13^C spectra were measured on a Bruker AVANCE 400 (400 MHz) spectrometer using TMS as the internal standard. Elemental analyses were measured on a HERAEUS (CHNO, Rapid) analyzer. IR spectra were recorded on a Bio-Rad FTS-40 spectrometer (KBr). Sonication was performed in Shanghai Branson-CQX ultrasonic cleaner (with a frequency of 25 kHz and a nominal power 250 W) and SK 250 LH ultrasonic cleaner (with a frequency of 40 kHz, 59 kHz and a nominal power 250 W; Shanghai Kudos Ultrasonic Instrument Co., Ltd.). The reaction flask was located in the cleaner, where the surface of reactants is slightly lower than the level of the water. The reaction temperature was controlled by addition or removal of water from ultrasonic bath.

Syntheses

The preparation of chalcones was referred to Ref.. [15]

Chalcones (**1,** 2 mmol), phenylhydrazine hydrochloride (**2,** 6 mmol) and sodium acetate (0.3 mmol) were dissolved in acetic acid aqueous solution (6 mL, HAc/H_2_O=2/1,V/V ) in a 50 mL conical flask. The mixture was irradiated in the water bath of an ultrasonic cleaner for the period as indicated in Table . The reaction mixture was poured into crushed ice. The precipitate was separated by filtration, washed with water, and crystallized from ethanol to obtain the 1,3,5-triaryl-2-pyrazolines. The authenticity of **3c, 3d, 3f** was established by comparing their melting point with literature. [7,9] The rest were established by their ^1^H NMR, ^13^C, IR and elemental analysis data.

**3a** ^1^H NMR (CDCl_3_): *δ *3.14 (dd, J=7.2, 17.2 Hz, 1H), 3.80 (s, 3H, OCH_3_), 3.84 (dd, J=12.4, 16.8 Hz, 1H), 5.26 (dd, J=7.2, 12 Hz, 1H) 6.80-7.75 (m, 14H) ppm. Anal. calcd. for C_22_H_20_N_2_: C 84.62, H 6.41, N 8.97; found C 84.60, H 6.44, N 8.99. ^13^C NMR: 44.03, 55.67, 64.44, 113.82, 114.37, 119.45, 126.32, 127.93, 128.50, 128.94, 129.27, 130.46, 133.24, 135.07, 145.31, 147.11. IR (KBr) *ν* _max_* *:1115，1261，1504，1598cm^-1^.

**3b** ^1^H NMR (CDCl_3_): *δ *2.38 (s, 3H, CH_3_), 3.15 (dd, J=7.2, 17.2 Hz, 1H), 3.85 (dd, J=12.4, 17.2 Hz, 1H), 5.27 (dd, J=6.8, 12 Hz, 1H) 6.77-7.75 (m, 14H) ppm. Anal. calcd. for C_22_H_20_N_2_: C 84.62, H 6.41, N 8.97; found C 84.61, H 6.43, N 9.00. ^13^C NMR: 21.68, 44.04, 64.71, 113.78, 119.43, 126.28 128.43, 128.90, 129.28, 129.60, 130.16, 133.07, 137.63, 141.51, 145.38, 147.10. IR (KBr) *ν* _max_* *:1112，1499，1594cm^-1^.

**3e** ^1^H NMR (CDCl_3_): *δ * 3.08 (dd, J=6.8, 17.2 Hz, 1H), 3.40 (dd, J=12.4, 17.2 Hz, 1H), 5.68 (dd, J=6.8, 12.4 Hz, 1H) 6.83-7.77 (m, 14H) ppm. Anal. calcd. for C_21_H_17_N_2_: C 85.85, H 5.72, N 9.43; found C 85.83, H 5.73, N 9.45. ^13^C NMR: 42.33, 61.57, 113.55, 119.63, 124.49, 127.77, 128.03, 128.96 129.13, 129.44, , 130.63, 132.50, 133.01, 139.62, 144.85, 147.50. IR (KBr) *ν* _max_* *:1122,1506, 1599 cm^-1^.

**3g** ^1^H NMR (CDCl_3_): *δ * 3.06 (dd, J=6.8, 17.1 Hz, 1H), 3.37 (dd, J=12.4, 17.2 Hz, 1H), 5.65 (dd, J=6.8, 12.4 Hz, 1H) 6.81-7.76 (m, 14H) ppm. Anal. calcd. for C_21_H_17_N_2_: C 85.85, H 5.72, N 9.43; found C 85.81, H 5.73, N 9.44. ^13^C NMR: 42.31, 60.43, 113.35, 119.52, 124.19, 127.99, 128.03 129.01, 129.19, 129.32, 130.57, 132.44, 133.18, 139.62, 145.29, 147.11. IR (KBr) *ν* _max_* *:1118, 1502, 1599 cm^-1^.

**3i** ^1^H NMR (DMSO): *δ * 3.14 (dd, J=7.2, 17.2 Hz, 1H), 3.84 (dd, J=12.4, 17.2 Hz, 1H), 5.32 (dd, J=7.6, 12.4 Hz, 1H) 6.80-7.68 (m, 14H) ppm. Anal. calcd. for C_21_H_17_N_2_: C 84.85, H 5.72, N 9.43; found C 84.87, H 5.74, N 9.46. ^13^C NMR: 42.34, 61.37, 113.50, 117.21, 126.81, 126.90, 128.60, 129.11, 129.36, 130.60, 132.13, 132.19, 136.01, 139.60, 143.80, 147.33. IR (KBr) *ν* _max_* *:1120, 1501, 1595cm^-^
